# Molecular epidemiology of respiratory viruses in virus-induced asthma

**DOI:** 10.3389/fmicb.2013.00278

**Published:** 2013-09-12

**Authors:** Hiroyuki Tsukagoshi, Taisei Ishioka, Masahiro Noda, Kunihisa Kozawa, Hirokazu Kimura

**Affiliations:** ^1^Gunma Prefectural Institute of Public Health and Environmental SciencesGunma, Japan; ^2^Infectious Disease Surveillance Center, National Institute of Infectious DiseasesTokyo, Japan

**Keywords:** molecular epidemiology, virus-induced asthma, respiratory syncytial virus, human rhinovirus, human metapneumovirus, respiratory viruses

## Abstract

Acute respiratory illness (ARI) due to various viruses is not only the most common cause of upper respiratory infection in humans but is also a major cause of morbidity and mortality, leading to diseases such as bronchiolitis and pneumonia. Previous studies have shown that respiratory syncytial virus (RSV), human rhinovirus (HRV), human metapneumovirus (HMPV), human parainfluenza virus (HPIV), and human enterovirus infections may be associated with virus-induced asthma. For example, it has been suggested that HRV infection is detected in the acute exacerbation of asthma and infection is prolonged. Thus it is believed that the main etiological cause of asthma is ARI viruses. Furthermore, the number of asthma patients in most industrial countries has greatly increased, resulting in a morbidity rate of around 10-15% of the population. However, the relationships between viral infections, host immune response, and host factors in the pathophysiology of asthma remain unclear. To gain a better understanding of the epidemiology of virus-induced asthma, it is important to assess both the characteristics of the viruses and the host defense mechanisms. Molecular epidemiology enables us to understand the pathogenesis of microorganisms by identifying specific pathways, molecules, and genes that influence the risk of developing a disease. However, the epidemiology of various respiratory viruses associated with virus-induced asthma is not fully understood. Therefore, in this article, we review molecular epidemiological studies of RSV, HRV, HPIV, and HMPV infection associated with virus-induced asthma.

## INTRODUCTION

Acute respiratory illness (ARI) is a major cause of morbidity and mortality worldwide (Williams et al., 2002; Sloots et al., 2008). ARI imposes a large burden on health, particularly in children. For community-based care, ARI has been estimated at a cost of over US$100 per case (Ehlken et al., 2005). The disease burden for ARI is estimated at 94,037,000 disability-adjusted life years and 3.9 million deaths (World Health Organization, 2002). Thus, ARI has a huge impact on health and society.

Although severe lower respiratory tract infections have been observed, ARI is most often associated with mild upper respiratory infection (URI). Most ARI cases in early childhood are confirmed as URI, leading to symptoms of the common cold with coryza and cough. In contrast, around one-third of infants with ARI develop lower respiratory tract symptoms such as tachypnea, wheezing, severe cough, breathlessness, and respiratory distress (Tregoning and Schwarze, 2010). In general, viruses are the most common causative agents of ARI. More than 200 different types of viruses are known to cause ARI, with respiratory syncytial virus (RSV), human rhinovirus (HRV), human metapneumovirus (HMPV), and human parainfluenza virus (HPIV) most commonly identified in ARI patients. Indeed, together with these respiratory viruses, human enterovirus (HEV), influenza virus (InfV), human coronavirus (HCoV), adenovirus (AdV), and human bocavirus (HBoV) account for around 70% of ARIs detected (Kusel et al., 2006). Respiratory viral infections can have severe adverse outcomes in patients with established asthma and are associated with nearly 80% of asthma exacerbation episodes (Nicholson et al., 1993; Johnston et al., 1995; Wark et al., 2002; Heymann et al., 2004; Grissell et al., 2005). Accumulating evidence indicates that the etiology of most cases of asthma, namely virus-induced asthma, is linked to such respiratory virus infections. In addition, RSV and HRV are the most frequently detected pathogens and may play an important role in viral induction and exacerbation of asthma.

Molecular biology techniques have developed rapidly over recent years. The application of molecular techniques to the study of virus-induced asthma enhances epidemiologic studies by improving our ability to classify these pathogens into meaningful groups (Foxman and Riley, 2001). In this review, we focus on molecular epidemiological studies of respiratory viruses, including RSV, HRV, HMPV, and HPIV infections, associated with virus-induced asthma.

## VIRAL INFECTION AND ASTHMA

In infancy, illnesses such as bronchiolitis share many clinical features with acute asthma, including wheezing, rapid breathing, prolonged expiratory phase inflammation, and respiratory compromise. Respiratory viruses are detected in the majority of asthma exacerbations in both children (80–85%) and adults (75–80%; Johnston et al., 1995; Grissell et al., 2005). In addition, wheezing illnesses are also closely associated with respiratory viral infections in all age groups (Gern, 2010). Fujitsuka et al. (2011) attempted to detect various respiratory viruses in Japanese children with acute wheezing using PCR technology and found viruses in samples from 86.1% patients: RSV or HRV alone were detected in 40.9 and 31.3% patients, respectively and both RSV and HRV were detected in 12.2% patients. Other previous reports suggested that the prevalence of RSV and HRV is similar (36 and 42%, respectively) in children less than 2 years of age, but differs (27 and 66%) in older children (Johnston et al., 1995; Grissell et al., 2005). In addition, Fujitsuka et al. (2011) suggested that RSV was the dominant species detected in patients with no history of wheezing and/or asthma, while HRV was dominant in patients with such a history. Thus, the main causative viral agent of asthma depends on previous illness and age.

**FIGURE 1:**
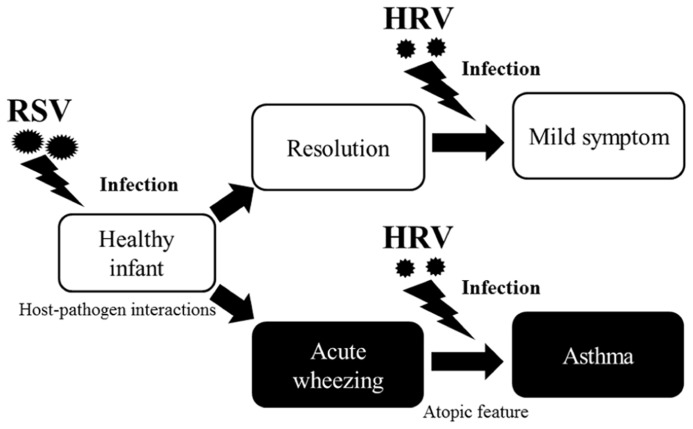
**Relationship between respiratory viral infections and development of asthma.** Host-pathogen interactions that determine the severity of respiratory illnesses, and risk for subsequent asthma was increased by respiratory virus infection, especially due to RSV, in infants. Although most acute wheezing resolves within a relatively short time, a history of wheezing and host immunological conditions (e.g., atopic features) heightens the risk for asthma. Once asthma is established, HRV infections are the most common causative agents of asthma in children.

Around one-third of infants who have an acute wheezing illness go on to develop recurrent wheezing, indicating that viral respiratory illnesses in early life promote asthma. Recently, the “two-hit” hypothesis has been proposed, whereby viral infections promote asthma mainly in predisposed children (Gern, 2010). Infants who develop virus-induced wheezing episodes are at increased risk for subsequent asthma, but most acute wheezing illnesses in infancy resolve with no long-term sequelae. It has been recognized for years that RSV infections often produce the first episode of wheezing in children who go on to develop chronic asthma (Lemanske, 2004). Indicators of heightened risk for developing asthma include wheezing episodes caused by HRV infections and the development of atopic features such as atopic dermatitis, allergen-specific IgE for foods or aeroallergens (e.g., house dust, mites, or cat or dog dander), and blood eosinophilia (**Figure 1**). Once asthma has been established, HRV infections are the most common cause of acute exacerbations, especially in children. As in infancy, atopy is an important risk factor for acute episodes of virus-induced wheezing (Kusel et al., 2007). Many previous reports have suggested that such respiratory virus infections are deeply associated with virus-induced asthma (Kusel et al., 2007; Pierangeli et al., 2007; Kuehni et al., 2009; Fujitsuka et al., 2011; Kato et al., 2011). Thus, it is entirely plausible that viral infections induction and/or exacerbation asthma in children.

## MOLECULAR EPIDEMIOLOGY OF RSV

Respiratory syncytial virus of genus *Pneumovirus* and family *Paramyxoviridae* causes ARI in children (Vardas et al., 1999; Peter and James, 2006). RSV infection may cause major problems in infants less than 1 year of age and can lead to life-threatening ARIs such as bronchiolitis and bronchopneumonia (Shay et al., 1999; Leung et al., 2005; Yorita et al., 2007). Epidemiological studies suggest that around 70% of infants have experienced an RSV infection by the age of 1 year, and 100% by the age of 2 years; host response to the virus varies greatly, but includes upper respiratory tract infections, typical bronchiolitis, and RSV-induced wheezy bronchitis (Cane, 2001; Kuehni et al., 2009). Long-term prospective case-control and cohort studies have also linked RSV bronchiolitis to the development of wheezing and asthma later in childhood (Sigurs et al., 1995, 2005, 2010; Henderson et al., 2005). Thus, RSV infections may be associated with the initiation and/or exacerbation of asthma.

The RSV genome encodes 11 proteins (Peter and James, 2006). Among these, the attachment glycoprotein (G) is a major structural protein that may be associated with both infectivity and antigenicity (Johnson et al., 1987; Rueda et al., 1991). Molecular epidemiological studies have shown that RSV can be classified into two phylogenetic subgroups, RSV-A and RSV-B (Mufson et al., 1985). The strains of subgroup A can be subclassified into eight genotypes (GA1–GA7 and SAA1), as can those of subgroup B (BA, GB1–GB4, and SAB1–3; Parveen et al., 2006). From phylogenetic analysis of the *G* gene of RSV, Martinello et al. (2002) showed that RSV belonging to GA3 genotype may be associated with greater severity of illness in, for example, bronchiolitis and pneumonia. Although GA3 genotype has been detected in the United Kingdom, Spain, and New Zealand, it is not the most prevalent strain (Cane et al., 1991; Garcia et al., 1997; Matheson et al., 2006). Martinello et al. (2002) therefore suggested that the association between greater severity of illness and GA3 genotype may be solely due to a transient shift in genotype-specific immune status within the community. In addition, correlations between certain strains and/or genotypes of RSV and slight differences in disease severity have been described previously (Hall et al., 1990; Walsh et al., 1997). Some genotypes such as subgroup A genotypes GA1, GA2, GA5, GA7, and NA1 and subgroup B genotype BA have been detected throughout the world in recent years (Zlateva et al., 2004; Parveen et al., 2006; Zhang et al., 2007; Nakamura et al., 2009; Rebuffo-Scheer et al., 2011). Of these, NA1 is a novel genotype known to be genetically close to GA2 genotype, while GA2 genotype and BA genotype are the most common genotypes of RSV subgroups A and B around the world and have persisted for many years (Tran et al., 2013). Furthermore, a new genotype belonging to RSV-A, ON1, has been detected in some countries, including Canada, Korea, Malaysia, South Africa, and Japan (Eshaghi et al., 2012; Lee et al., 2012; Khor et al., 2013; Tsukagoshi et al., 2013; Valley-Omar et al., 2013). This genotype contains a unique tandem repeat (72nt sequence duplication) in the C-terminal 3rd hypervariable region of the *G* gene, and may be classified as a subdivision of NA1 (Eshaghi et al., 2012). Some reports have suggested that the severity of illness is not linked to subgroups or genotypes, but is associated with the quantity of RSV in nasopharyngeal aspirate (Sullender, 2000; Campanini et al., 2007). A larger population study is needed to identify the different RSV genotypes circulating in different areas to gain a better understanding of the relationship between disease severity and RSV genotype.

The G protein is a major antigen of RSV and amino acid substitutions may be related to changes in antigenicity. There are some reports of amino acid substitutions, and some positively selected sites in the C-terminal 3rd hypervariable region of the *G* gene have been estimated (Botosso et al., 2009; Yoshida et al., 2012; Kushibuchi et al., 2013). For example, Yoshida et al. (2012) estimated some sites under positive selection in the region (Asn250Ser, Met262Glu, Arg297Lys,and Arg297Glu substitutions in RSV-A strains were estimated by the REL method, and Asn273Tyr and Leu274Pro substitutions of RSV-A, as well as Leu237Pro substitution of RSV-B, were estimated by the IFEL method). Botosso et al. (2009) found 29 and 23 amino acid sites under putative positive selection in RSV-A and RSV-B, respectively. In addition, some unique positively selected sites were found in the *G* gene (Kushibuchi et al., 2013). These amino acid variations at these sites might play a key role in severe respiratory infection, such as bronchiolitis (Goto-Sugai et al., 2010). Furthermore, the rate of molecular evolution of the region might be high. For example, Kushibuchi et al. (2013) estimated the evolutionary rate of RSV-A at 3.63 × 10^-^^3^ substitutions/site/year, while that of RSV-B was estimated at 4.56 × 10^-^^3^ substitutions/site/year. Thus, it is suggested that this C-terminal 3rd hypervariable region in the *G *gene of RSV-A and -B evolved rapidly (Kushibuchi et al., 2013). Based on host immunological conditions, it is suggested that host immunity such as TLR4 polymorphism is linked to symptomatic RSV infection (Delgado et al., 2009). Thus, both the antigenicity of the viruses and host immune conditions may play important roles in the pathophysiology of severe respiratory infections such as bronchiolitis, pneumonia, and virus-induced asthma (Awomoyi et al., 2007).

## MOLECULAR EPIDEMIOLOGY OF HRV

Human rhinovirus are a group of positive-sense ssRNA viruses belonging to genus *Enterovirus* in the family *Picornaviridae* (Turner and Couch, 2007). Although HRVs were previously thought to be mainly associated with the common cold causing mild respiratory symptoms, recent reports strongly suggest that HRVs may induce and/or exacerbate asthma (virus-induced asthma; Chung et al., 2007; Turner and Couch, 2007; Busse et al., 2010; Gern, 2010; Khadadah et al., 2010). One report suggested that HRV wheezing illness within the first three years of life is significantly associated with the development of asthma at age 6 years (Jackson et al., 2008). Another report suggested that HRVs are major agents in the induction of wheezing and exacerbation of asthma (Khadadah et al., 2010). Thus, HRVs are being re-evaluated as important agents of ARI in humans (Imakita et al., 2000; Papadopoulos et al., 2002; Wos et al., 2008). The basis for these lower respiratory symptoms has been a source of controversy in terms of the mechanisms of HRV pathogenesis. There are a variety of potential barriers to HRV infection of the lungs, including temperature-sensitive replication of the virus. For this reason, it is thought that the optimum propagation temperature of HRVs may be 32–35°C *in vitro* (Papadopoulos et al., 1999; Schroth et al., 1999). However, a recent study suggested that HRVs can propagate in lower airway tissues and this may be an important factor in the development of airway obstruction, coughing, and wheezing that can lead to bronchiolitis and pneumonia (Mosser et al., 2005). HRV has been concomitantly isolated with bacterial pathogens in 24–54% of children and 10–18% of adults with pneumonia (Juven et al., 2004; Templeton et al., 2005; Jennings et al., 2008). Thus, it is not clear whether HRV is ever the causative agent for the disease.

Human rhinovirus were previously classified into two species, HRV species A (HRV-A) and species B (HRV-B), containing over 100 serotypes (Turner and Couch, 2007). However, a genetically heterogeneous third species, HRV species C (HRV-C), was discovered recently (Lamson et al., 2006; McErlean et al., 2007). Recent reports suggest that HRV-A, B, and C have a unique and wide genetic diversity (McIntyre et al., 2010; Simmonds et al., 2010; Arakawa et al., 2012). HRV-A and -C appear to be mainly associated with ARIs and virus-induced asthma, while HRV-B has been detected in a relatively small number of patients with ARIs (Linsuwanon et al., 2009; Wisdom et al., 2009; Smuts et al., 2011). Our previous findings obtained from samples from children with ARIs in Japan indicated that HRV-A and -C can be classified into many clusters in the phylogenetic tree, with 30% nucleotide divergence of the *VP4/VP2* coding region (Mizuta et al., 2010a; Arakawa et al., 2012; Kiyota et al., 2013). In addition, Kiyota et al. (2013) estimated that the rate of molecular evolution of the *VP4/VP2 *coding region was rapid (3.07 × 10^-^^3^ substitutions/site/year) in HRV-C. These results suggest that HRV-A and -C detected in ARI cases are the predominant strains and have varied genetic properties (Wisdom et al., 2009; Mizuta et al., 2010a; Arakawa et al., 2012). Thus, the association between HRV type and disease severity is not fully understood. There may be important differences in the susceptibility of individuals to the replication of HRV in lower airway tissues.

Parry et al. (2000) and Gern et al. (2000) found that weak peripheral blood mononuclear cell (PBMC) Th1 (IFN-γ) response to HRV infection is associated with increased viral shedding, and decreased proliferative response of PBMCs to HRV is associated with increased severity of symptoms. In addition, it was found that weak Th1 responses (IFN-γ/IL-5 mRNA ratio) in sputum are also associated with greater severity of illness (Gern et al., 2000). Furthermore, weak Th1 responses to viral infection in adults with asthma have been associated with decreased lung function and greater airway responsiveness (Brooks et al., 2003). These results indicate that individuals with a weak Th1 response to viruses, and perhaps individuals with asthma in general, may be more susceptible to HRV illnesses, and this association may be strongest in those with more severe disease (Parry et al., 2000; Gern et al., 2000; Brooks et al., 2003). Other epidemiological and biological factors, such as allergy, atopic dermatitis, or a family history of allergy, may be related to virus-induced asthma (Green et al., 2002; Singh et al., 2007). Recently it is suggested that variants at the 17q21 locus were associated with HRV induced asthma in children who had a history wheezing illnesses, although associations of 17q21 variants with asthma were restricted to children who had a history of HRV wheezing illnesses (Calişkan et al., 2013).

## MOLECULAR EPIDEMIOLOGY OF HMPV

Human metapneumovirus is a recently identified RNA virus belonging to the *Paramyxoviridae* family, of genus *Metapneumovirus* (Collins and Crowe, 2007). HMPV is a major pathogen that causes ARI in all ages (Collins and Crowe, 2007). The first HMPV infection appears to take place within the first six months of life, after which infections may occur repeatedly and frequently (Schildgen et al., 2011). The nosocomial impact of HMPV is estimated to be as high as that for RSV. In an HMPV outbreak in Japan, 34.8% of elderly patients who shared the same day care room in a hospital were infected with HMPV (Honda et al., 2006). Higher morbidity is observed in young children, the elderly, and immunocompromised adults (Boivin et al., 2002; Falsey et al., 2003; van den Hoogen et al., 2003; Sumino et al., 2005; Williams et al., 2005; O’Gorman et al., 2006). HMPV is classified into two genotypes (A and B) and four subgroups (A1, A2, B1, and B2) by phylogenetic analysis, using the *F* and *G* genes (Biacchesi et al., 2003; van den Hoogen et al., 2004). Subgroup A2 has been subdivided into two lineages, subgroup A2a and A2b (Huck et al., 2006). It has been suggested that these genotypes circulate in variable proportions in some areas (Gerna et al., 2005; Mackay et al., 2006). Although the molecular epidemiological information on HMPV has gradually accumulated, the detailed epidemiology remains unclear (Mizuta et al., 2010b; Pitoiset et al., 2010; Omura et al., 2011). HMPV infections can occur throughout the year, but seasonality has been described in several studies, with the epidemiological peak occurring several months later than that observed for RSV epidemics (Robinson et al., 2005; Wilkesmann et al., 2006; Madhi et al., 2007; Aberle et al., 2008, 2010; Heininger et al., 2009). It remains unclear whether different HMPV subgroups are associated with differences in the clinical course of disease. Several groups have suggested that HMPV subgroup A might be associated with more severe clinical disease (Martinello et al., 2002; Kaida et al., 2006; Vicente et al., 2006; Arnott et al., 2013), while others have reported that subgroup B may cause more severe illness (Esper et al., 2004; Pitoiset et al., 2010), and still other groups have found no evidence for differential severity caused by different HMPV lineages (Agapov et al., 2006; Manoha et al., 2007; Larcher et al., 2008; Xiao et al., 2010). Previous reports suggested that the substitution rates for the *G* gene (3.5 × 10^-^^3^ substitution/site/year) and the *F* gene (7.1 × 10^-^^4^ to 8.5 × 10^-^^4^ substitution/site/year) are high, and some positively selected sites have been found in the latter (de Graaf et al., 2008; Yang et al., 2009). It may be that there is a correlation between some positively selected epitopes and disease severity. Thus, the association between HMPV subgroup and disease severity is controversial. To gain a better understanding of host responses that may contribute to differences in clinical severity between HMPV subgroups, a more detailed analysis that includes host immunological status is needed.

## MOLECULAR EPIDEMIOLOGY OF HPIV

Human parainfluenza virus belong to the *Paramyxoviridae* family. There are two genera of HPIV, *Respirovirus* (HPIV-1 and HPIV-3) and *Rubulavirus* (HPIV-2 and HPIV-4; Karron and Collins, 2007). HPIV is classified into four serotypes (HPIV1–4), all of which can cause various ARI in humans such as URI, croup, bronchitis, asthma, and pneumonia (Henrickson, 2003; Karron and Collins, 2007). Although HPIV type 4 (HPIV4) is rarely reported, HPIV1-3 are important causes of various ARI, including the common cold, croup, bronchitis, bronchiolitis, and pneumonia in children, and they commonly re-infect both children and adults. While such infections are generally mild in healthy persons, they may cause serious diseases in children, such as asthma (Henrickson, 2003; Karron and Collins, 2007). Although fewer HPIV strains have been detected compared with other respiratory viruses such as RSV, HRV, and HMPV, previous reports suggest that HPIV1 and 3 are the dominant viruses in children with ARI (Reed et al., 1997). Indeed, serological surveys indicate that at least 60% of children have been infected with HPIV3 by 2 years of age, approximately 80% have been infected by age 4, and at least 75% have been infected with HPIV1 by 5 years of age (Parrott et al., 1959, 1962). HPIV1 and 3 show high prevalence and are associated with up to 12% of acute lower respiratory tract infections in adults (Azevedo et al., 2003; Matsuse et al., 2005). HPIV1 and HPIV3, may be major agents of ARI throughout the world, along with other viruses such as RSV, HRV, and HMPV (Laurichesse et al., 1999; Iwane et al., 2004; Monto, 2004; Do et al., 2011). In addition, it is suggested that HPIV is a major causative agent of virus-induced asthma (Henrickson and Savatski, 1997). Several previous studies have reported that HPIV1 infections demonstrate clear outbreaks in autumn, mostly in September and November, every 2 years (Knott et al., 1994; Hall, 2001; Counihan et al., 2001). Other studies have reported that HPIV3 causes yearly outbreaks around the globe, mainly in the spring-summer season (Knott et al., 1994; Counihan et al., 2001; Hall, 2001; Mizuta et al., 2013). A recent study suggested that four different types of HPIV cause similar clinical manifestations in patients, and the clinical presentation of HPIV infection may differ depending on patient age (Liu et al., 2013).

Henrickson and Savatski (1996) analyzed the longitudinal evolution of the HN coding region in 13 strains of HPIV1 isolated in the USA. These results showed that the antigenic and genetic subgroups are very stable. In addition, Mizuta et al. (2011) suggested that the evolution of the *HN* gene in the present HPIV1 isolates was relatively slow and that the gene is highly conserved. Only a few reports on the molecular epidemiology of HPIV1 are available and it appears that the molecular epidemiology of HPIV is poorly understood. Larger and more detailed studies on the association of HPIV with asthma are needed.

## MOLECULAR EPIDEMIOLOGY OF OTHER VIRUSES

HEV68 was recently detected in asthmatic patients (Hasegawa et al., 2011). HEV68 was found to be relatively acid resistant and thus could be distinguished from acid-sensitive HRV87 (Schieble et al., 1967; Kapikian et al., 1971). HRV87 was recently reclassified as HEV68 based on phylogenetic analysis and neutralization test, and some laboratories have confirmed its acid sensitivity (Blomqvist et al., 2002; Ishiko et al., 2002; Savolainen et al., 2002). Distinguishing between HRV and HEV based on the acid sensitivity of isolates is therefore not appropriate for HEV68. The number of reports of an association between respiratory disease and HEV68 infection has recently increased. One report of the phylogenetic analysis of HEV68 based on partial VP1 gene sequences indicates wide genetic diversity (Linsuwanon et al., 2012). In addition, Tokarz et al. (2012) showed the presence of multiple clades among the circulating strains, and that all strains are spreading rapidly worldwide and contributing to the prevalence rates of respiratory diseases. In addition, asthmatic individuals infected with HEV68 also have the propensity to develop unstable asthma or an acute attack (Hasegawa et al., 2011).

Influenza virus is also a major causative agent of ARI in both children and adults. Furthermore, asthmatic patients were found among children and adults hospitalized with seasonal InfV (Dao et al., 2010; Dawood et al., 2010). Although it is recognized that viral infections such as RSV or HRV may induce and/or exacerbate asthma, the effect of InfV on asthma remains arguable (Johnston et al., 1995). Although one study suggested that A(H1N1)pdm09 viruses impose greater risk factors on children than seasonal InfV (Tran et al., 2012), InfV vaccine was available before the influenza season since InfV causes more severe illness than other respiratory viruses. Therefore, it is suggested that InfV vaccine be recommended for children with asthma (Kloepfer et al., 2012).

Although the level of detection of HCoV, HBoV, or AdV is relatively low, these infections are also detected in children with acute wheezing (Chung et al., 2007; Jartti et al., 2007). Further studies are needed to clarify the clinical roles of HCoV, HBoV, or AdV infections and those of other respiratory viruses. In particular, the prevalence of HCoV, HBoV, or AdV infection in healthy control subjects, assessment of disease severity by other clinical variables, and the immunological effects should be investigated.

## MOLECULAR EPIDEMIOLOGY OF CO-INFECTION

Infants with severe bronchiolitis have an increased risk of developing recurrent wheezing later in life (Chung et al., 2007). HRV may be detected concurrently with other viruses such as RSV, HMPV, InfV, or HCoV (Richard et al., 2008; Fujitsuka et al., 2011). Considering their ubiquity, it is interesting that the number of respiratory viruses detected concurrently with HRV strains is relatively low (Lambert et al., 2007; Mackay, 2007), supporting the concept that HRVs have a direct role in the clinical outcome of infection (Miller et al., 2007). In fact, HRV strains are co-detected with other pathogens in reproducible, but clinically undefined, patterns (Brunstein et al., 2008). The HRV partnership with host immunity may be a mutualistic one, inadvertently imparting an advantage to the host by protecting against more cytopathic respiratory viral pathogens while the host provides a vessel for HRV replication and transmission.

Respiratory viruses other than RSV and multiple viral infections may contribute to the severity of bronchiolitis and asthma. Indeed, it was reported that dual infections of HMPV and RSV or HRV and RSV confer a 5- to 10-fold increase of severe disease in children admitted to pediatric intensive care units (Papadopoulos et al., 2002; Semple et al., 2005). In contrast, other studies reported that co-infection with two respiratory viruses was not significantly associated with disease severity (van Woensel et al., 2006; Wolf et al., 2006). Thus, there is no consensus on the effects of co-infection on disease severity. The effect of dual infection may depend upon which viruses co-infect together. For example, although there was no increase in severity when HRV and/or AdV were detected during RSV infection, co-infection with both HMPV and RSV increased the rate of intensive care unit admissions (Aberle et al., 2005; Semple et al., 2005). Thus, although dual infections and reinfections have been well documented in children, chronic infection with the development of quasispecies cannot be ruled out without obtaining more complete data using high performance detection methods (Hall and McCarthy, 1996).

## CONCLUSION

Respiratory viral infections are a major cause of virus-induced asthma in early life. Although antiviral therapy is not yet available for patients infected with respiratory viruses, the detection and identification of these viruses could help to explain serious respiratory illness, provide guidance for medical care, and prevent unnecessary treatment with antibiotics. Based on the results of many related studies, we propose a two-step hypothesis of asthma development in children. The first step is mainly due to RSV infection: when RSV infects bronchial cells, the bronchial cells produce various cytokines and chemokines. These responses cause hyperresponsiveness in the bronchial cells. In other words, RSV infection might create a preparatory step as the first step in the development of asthma. HRV infection might then bring about the second step in the development of asthma. An infant with a history of wheezing caused by RSV infection may develop the heavy wheezing of asthma due to HRV infection followed by RSV infection. To understand the cause of asthma, we need to examine the larger complex picture of genetic susceptibility, immune components, environmental exposures, and the interactions between these elements.

## Conflict of Interest Statement

The authors declare that the research was conducted in the absence of any commercial or financial relationships that could be construed as a potential conflict of interest.
